# The change in the clinical presentation of Graves’ disease: a 30 years retrospective survey in an academic Brazilian tertiary center

**DOI:** 10.20945/2359-3997000000265

**Published:** 2020-06-19

**Authors:** Wanesa Pinto, João H. Romaldini, Nicolas Perini, Roberto B. Santos, Danilo Villagelin

**Affiliations:** 1 Universidade Estadual de Campinas Campinas SP Brasil Curso de Pós-Graduação em Medicina Interna, Universidade Estadual de Campinas (Unicamp), Campinas, SP, Brasil; 2 Hospital Pontifícia Universidade Católica de Campinas Campinas SP Brasil Endocrinologia e Metabolismo, Hospital da Pontifícia Universidade Católica de Campinas (PUC-Campinas), Campinas,SP, Brasil

**Keywords:** Graves’ disease, Graves’ orbitopathy, hyperthyroidism

## Abstract

**Objective:**

Graves’ disease (GD) is the main cause of hyperthyroidism among adults. It is an autoimmune condition classically marked by the Merserburg Triad (goiter, thyrotoxicosis, and orbitopathy), but the change in presentation of GD over time has rarely been studied. To determine changes in the clinical presentation of patients with GD in the last 30 years.

**Subjects and methods:**

The study evaluated 475 patients diagnosed with GD between 1986 and 2016 in a single center. Patients were evaluated regarding epidemiological aspects, thyroid function, inflammatory activity of the eyes evaluated by the Clinical Activity Score; CAS, severity evaluated by NOSPECS classification and thyroid volume estimated by ultrasonography.

**Results:**

Patients assessment identified an increase in the mean age of diagnosis of GD (p < 0.02), a reduction in thyroid volume (p < 0.001) and less intense orbital involvement from 2007-2016 compared to 1986-2006 (p = 0.04). The number of smoking patients was smaller from 2007 to 2016 (28.7%) than 1986 to 2006 (42.8% p = 0.001). The TSH and TRAb values did not had significant changes.

**Conclusion:**

GD presentation appears to be changed in the last years compared to the typical initial presentation. There is a less frequent inflammatory involvement of orbital tissue, smaller goiters, a lower number of smokers and diagnosis at older age.

## INTRODUCTION

Robert James Graves was an Irish physician who described three women with goiter and palpitations during his lectures in 1835 ( 1 ). Later, an addition patient with exophthalmos was added ( 2 ). In 1825, Parry had already noted one patient developing goiter and palpitations post-partum. Before them, in 1802, the Italian physician Flajani had documented goiter and cardiac palpitations but without exophthalmos ( 2 ). Some years later, a German physician named Von Basedow described the coincidence of heart palpitations, exophthalmos, and goiter: the so-called Merseburg Triad ( 3 ).

Graves’ disease (GD) is currently the main cause of hyperthyroidism in young adults affecting around 0.5% of the global population ( 4 ). The majority of cases are seen in women aged 35 to 60, but the condition may manifest itself in any age ( 4 ). GD is caused by an interaction between genetic and environmental factors leading to the collapse of immune tolerance; of known environmental factors, smoking seems to be the most important ( 5 ). Other factors such as stress, infections, and pregnancy are correlated with disease development of GD ( 5 ). GD is initiated by the production of IgG autoantibodies directed against the TSH receptor (TRAb). These antibodies bind to and activate the receptor increasing production of thyroid hormones as well as local inflammatory reactions, glandular hyperplasia, and hypertrophy ( 6 , 7 ). TRAb is also an important factor in the development of Graves’ orbitopathy (GO) via the activation of orbital fibroblasts and production of inflammatory cytokine ( 6 ). The major risk factors for development and/or worsening of GO are severe hyperthyroidism, high TRAb values, and smoking ( 8 ). GO is characterized by inflammation and edema of extraocular muscles and an increase in the adipose and connective tissue of the orbit ( 9 ); in contrast, orbital edema is due to the hydrophilic action of glycosaminoglycans ( 10 ).The inflammatory reaction is attributed to the infiltration of extraocular muscles and connective tissue of the orbit by lymphocytes and macrophages. However, increased tissue volume is responsible for most of the manifestations of GO ( 7 ).

The clinical presentation of classically described GD include signs of thyrotoxicosis (palpitations, tremulousness, heat intolerance, weight loss, and anxiety) as well as tachycardia, tremor, proptosis, and thyroid enlargement on physical examination ( 5 ). Recent data from the literature suggest that the classical phenotype of GD may no longer apply to most patients. Bartalena and cols. in Italy described how relevant proportion of patients at diagnosis have mild to moderate GD. Nearly half of them have no goiter, and slightly less than one-fifth have subclinical hyperthyroidism; only 20% have GO ( 11 ). Therefore, the objective of this retrospective study was to evaluate the likely changes in GD phenotype in a large population of newly diagnosed patients in a single university hospital center.

## SUBJECTS AND METHODS

After institutional research ethics approval (number 66282417.5.0000.5463/Conep), all patients who were diagnosed with GD between 1986 and 2016 were retrospectively reviewed. The initial cohort study consisted of 567 patients seen at the Hospital PUC-Campinas from 1986 to 2016 with untreated GD; 92 patients were excluded: 58 patients had been treated before the initial evaluation in our center, and 34 patients did not have all data available or confirmed diagnosis of GD. The remaining group included 475 new GD patients. All hyperthyroid patients met the inclusion criteria: diagnosis of GD and hyperthyroidism characterized by diffuse goiter, increased serum levels of free T4, a suppressed serum TSH level, and the presence of positive serum TRAb and/or increased thyroidal uptake of Iodine 131 (until 2000 – reference values: 4 hours = 4-14% and 24 hours = 15-41%). After 2000 99mTc-pertechnetate thyroid uptake was used (reference values: 0.14%-0.16%).

Thyroid ultrasound was used to assess thyroid volume. The initial patients were evaluated by Toshiba ultrasonography (Otawara, Japan), from 1996 to 2000 the GE ultrasound model LOGIQ 500 (Tokyo, Japan) was used. After this period Toshiba ultrasound model SSA- 240A (Tokyo, Japan) was the ultra sound device used until 2010. In the last years GE model vivid T8 (Jiangsu, China) was incorporated.

The thyroid volume was calculated by multiplying the height, width, and depth of each lobe are measured and multiplied. The obtained result is then multiplied by a correction factor, which is π/6, or 0.524 ( 12 ). The normal value range of thyroid volume in our population is of 6 to 15 mL (the normal values of thyroid volume was performed through a sample of individuals without thyroid disease performed by the radiology department).

GO activity was estimated using the clinical activity score (CAS) according to Mourits and cols. ( 13 ) from 1992. There was one point for each of the following characteristics (ranging from 0 to 7): spontaneous retrobulbar pain, pain on attempted upwards or downwards gaze, redness of the eyelids, redness of the conjunctiva, swelling of the eyelids, inflammation of the caruncle, and conjunctival edema. Patients diagnosed between 1986 and 1991 CAS was not measured, for this reason these patients were excluded GO analyses. Intermittent and permanent diplopia was also evaluated; eyelid width was measured with a scale rule, and proptosis was measured using a Hertel exophthalmometer. GO severity was calculated by NOSPECS classification ( 14 ) data are evaluate only in 283 patients.

### Laboratory methods

Serum concentrations of TSH, was measured by an in-house solid phase microtiter RIA until 1986 with a sensitivity of 1.3 mU/mL and normal values < 8.3 mU/m ( 15 ). Then by an immunometric assay of 2^nd^ generation (TSHIRMA Serono kit) from 1987 to 1994 with a sensitivity of 0.01 mU/mL and normal values of 0.3-4.5 mU/mL. From 1995 to 2005 a 3^rd^ generation immunoenzymatic method was used (Abbott kit) with a sensitivity of 0.06 mU/mL and reference range of 0.32-5.2 mU/mL. After 2006 onwards a chemiluminescent assay, Immulite) has been used having larger sensitivity (0.002 mU/mL) and reference range of 0.3-4.1 mU/mL. Initially, Free T4 levels was estimated by the product of T3 resin uptake and total T4 levels and serves as a surrogate of the free hormone level with a normal range of 1.3-4.5. After 1994 Free T4 was measured by chemiluminescent assays (DPC Immulite system) and the normal range was 0.8-1.9 ng/dL. Before 1996 serum thyroglobulin antibody (TgAb), and thyroid peroxidase antibody (TPOAb) were measured by passive hemagglutination assay followed by IRMA method (normal values less than 200 mIU/mL and less than 150 mIU/mL, respectively), and then in 2005 by chemiluminescent assays (DPC Immulite system) with reference values positive > 40 IU/mL and > 35 IU/mL, respectively.

In the last 30 years we used some different methods for TRAb determinations. For instance from 1980 to 1991 we used an in-house assay which measures the TSAb activity of AMP cyclic stimulation in human thyroid plasma membrane ( 16 ). The TSAb positive reference was < 128%. From 1992 to 2008 we used first a radio-receptor assay (RSR Ltd) and then a TRAb 2^nd^ generation ELISA assay (RSR Ltd) with a normal range < 10 IU/L. Recently, we have used a human monoclonal assay (antibody M22) from Siemens Immunulite-TSI with normal values < 0.175 IU/L. To avoid confounders we express antithyroid antibodies as positive or negative.

### Statistical analysis

Statistical analyses were performed using SAS version 9.3 software (SAS Institute). Continuous variables with non-normal distribution were evaluated with the non-parametric tests. The Mann-Whitney test for medians and the Chi-squared test for proportions were used. To evaluate the trend of age at diagnosis, TSH, free T4, goiter volume, initial CAS, and longitudinal CAS values, were used with a linear regression model was used after the data were transformed in points. Finally, the Cochran-Armitage test evaluated gender and smoking over time. For all analyses, P < 0.05 was considered to be statistically significant.

## RESULTS

A total of 475 patients were analyzed, and 81% were female. The presence of TPOAb and TRAb were positive in about 70% (335 out 475) and 84% (342 out 404) of the cases, respectively. Regarding the treatment, most of the patients were treated with antithyroid drugs (mostly methimazole).

Table 1 shows details of the study patients who attended to thyroid clinic at the time of diagnosis. There was a trend to a higher percentage of patients diagnosed with GD after 2007 (58.7% against 41.3%). Between 1986 and 2006 (inclusive), the patients were younger and more patients were smokers. The subjects also had significantly larger goiters and higher CAS, but there was no difference in the proptosis or other clinical parameters.

**Table 1:** Clinical and laboratorial presentations of hyperthyroid Graves` disease patients in two different time period

Parameters			p-value
	1986-2006	2007-2016	
Number of patients	196	279	
Age (years)^a^	40.0 (33-49.1)	43.1 (33.1-52)	0.01
Gender, female^b^	145 (74%)	218 (78%)	0.21
Smoking positive	84 (42.8%)	80 (28.7%)	0.001
Thyroid volume (mL)^a^	25.65 (17.2-39.4)	18.2 (11.0-28.8)	0.001
Graves’ orbitopathy			
Clinical active score^a,d^	2.0 (1.0-3.0)	2.0 (1.0-2.5)	0.022
0	117 (60.3%)	188 (67.5%)	0.14
1	31 (15.9%)	47 (16.8%)	0.72
2	22 (11.3%)	22 (7,9%)	0.2.6
3	11 (5.6%)	11 (3.9%)	0.50
4	8 (4.1%)	6 (2.1%)	0.22
5	2 (1.0%)	5 (1.8%)	0.49
6	2 (1.0%)	0	
7	1 (0.5%)	0	
Clinical active score > 2	46 (23.4%)	44 (15.7%)	0.04
Proptosis (mm)	20.5 ± 1.9	21.3 ± 1.6	0.21
TSH (mlU/L)	0.025 ± 0.01	0.01 ± 0.008	0.66
Serum TRAb positive^e^	152 (91.8%)	190 (80%)	0.31
Serum TPOAb positive	133 (68.91%)	202 (71.63%)	0.53
Serum TgAb positive	81 (41.96%)	121 (42.90%)	0.85

^a^: Data are median and 25%-75% percentile.

^b^: Number and percentage in parenthesis.

^c^: Mean ± standard deviations.

^d^: Data available for n = 194 and n = 279 patients in each groups.

^e^: Data available for n = 166 and n = 238 patients in each groups.

Graves’ orbitopathy was of the most prominent eye.

TSH: thyrotropin; TRAb: thyroid receptor antibody.

p-value: Mann-Whitney test for means and Chi-squared test for proportions.

### Age at diagnoses

The median for ages were: 1986-2006: 40.0 years (25^th^-75^th^ percentile, 33.0-49.1 years) and significant higher in the group 2006-2016: 43.1 years (25^th^-75^th^ percentile, 33.1-52.0 years).

### Thyroid volume

**Figure 1:**
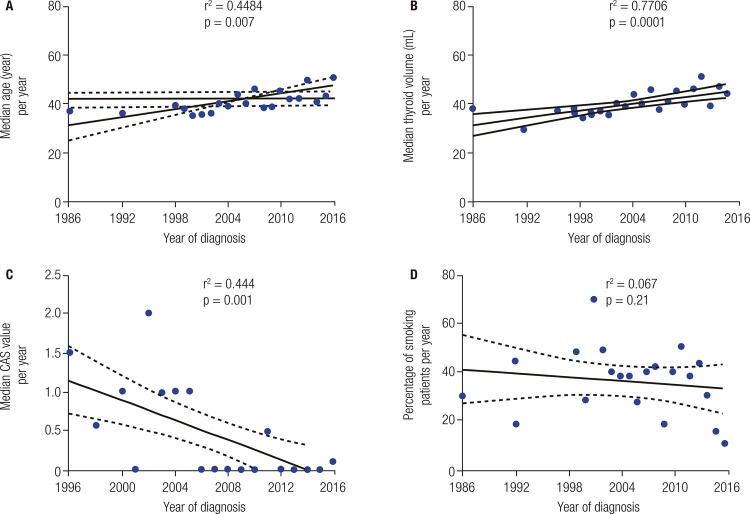
(A) Linear regression analysis of the median age of diagnosis in Graves’ disease patients. Data cover a 30-year period. (B) Linear regression analysis of the median thyroid volume (measured by ultrasonography) during the year of diagnosis among Graves’ disease patients. Data cover a 30-year period. (C) Linear regression analysis of the median clinical activity scores at diagnosis in Graves’ orbitopathy patients. Only patients with CAS ≥ 1 were included, data cover 20-year period. (D) Linear regression analysis of the estimated incidence (%) of smoking (in the year of diagnosis) among Graves’ disease patients. Dashed lines represent upper and lower 95% confidence intervals.

The median thyroid volume was 20.5 mL (25^th^-75^th^ percentile, 13.1-32.3 mL). It was significantly higher (25.65 mL; 25^th^-75^th^ percentile, 17.2-39.4 mL) from 1986 to 2006 ( Table 1 ) than between 2007 and 2016 (18.2 mL; 25th-75th percentile, 11.9-28.8 mL, p = 0.0001). Figure 1B showed that there was a significant reduction in the median thyroid volume (p < 0.0001) over time.

### Graves’ orbitopathy

The median of the CAS at diagnosis for the whole group was 2.0 (25^th^-75^th^, percentile, 1.0-3.0), and its distribution is shown in Table 1 ; 64% (n = 305) of the GD patients did not show signs of inflammatory activity in the orbit at diagnoses. Table 1 showed that the median CAS value was significantly (p = 0.022) lower (2.0; 25^th^-75^th^. percentile, 1.0-2.5) in the last period than the previous one (2.0; 25^th^-75^th^. percentile, 1.0-3.0). Figure 1C shows that the median GO score had a reduction over time suggesting a tendency towards minor inflammatory signs (p < 0.001).

Graves’ orbitopathy severity was available in 283 patients, 50% of patients from 1986-2006 and 70% of patients from 2007-2016. Comparing patients in group 1986-2006 and 2007-2016, the last group presented more frequently in class zero from NOSPECS classification (33 and 97 patients respectively, p < 0.001.) We found no differences in the other classes.

### Thyroid function

The mean serum TSH levels and the percentage of positive TRAb patients did not change over time.

### Smoking

Table 1 shows that the number of smoking patients was smaller from 2007 to 2016 (28.7%) compared to 1986 to 2006 (42.8% p = 0.001). This reduction was not significant after linear regression analysis (p = 0.21) over 30 years; however, there was a mild tendency to decline ( Figure 1D ).

## DISCUSSION

The literature has suggested that the classic clinical manifestation of GD (large goiters, intense orbitopathy and thyrotoxicosis) is perhaps no longer the most common phenotype in recent diagnosis ( 11 ).

A higher age at diagnosis correlates to with less symptomatic disease suggesting that GD patients in the 5^th^ decade of life have a clinical presentation with a less intense thyrotoxicosis symptoms and thus a more difficult diagnosis ( 17 , 18 ). Some data from fourth years ago suggested a peak for GO incidence from 25 to 45 years ( 18 ). Our population had an average age of diagnosis around the fourth decade of life.

Thyroid volume alterations are difficult to compare because not all studies used ultrasonography as the standard evaluation method. However, large goiters are less common in older age GD patients especially in older age groups or those with high iodine intake ( 19 ). Bartalena and cols. recently showed that smaller thyroid volumes may predominate in GD patients ( 11 ). Our study highlights the advantages of determining thyroid volume via ultrasound. This leads to more consistent data and further confirms the hypothesis of new clinical phenotypes for GD, which no longer include large goiters in the majority of patients.

GO is the main extrathyroidal manifestation of GD and may become an unusual disorder ( 19 ) following according to its decreasing in frequency in later years – especially severe forms. These news patterns of presentation may be challenging for physicians in the diagnosis of GD ( 20 ). The frequency of GO in GD patients has been shown to be different in the literature, and studies from 2012 suggest that orbital disease is present in about half of the patients with GD ( 21 ). In contrast, Rundle (1960) suggested that GO is present in 2/3 of the patients ( 22 ).

Data from the late 1980s and early 1990s showed a frequency from 13% to 48% ( 23 , 24 ) but these two studies evaluated proptosis rather than CAS. A review study showed that the GO presents clinical manifestations in only 25% to 50% of patients; however, 70% have signs of eye disease based on orbital imaging ( 25 ). On the other hand, smoking is probably the most important environment risk factor for GO ( 26 ). Some studies suggest that the effect of smoking in GO is dose-dependent with higher doses associated with more severe orbital inflammation and a higher risk of developing GO even among past smokers ( 26 ).

In the last decade – especially in western populations – an important reduction in GO was seen mainly as a result of antismoking legislation ( 27 ). We observed a decrease in tobacco use in Brazil (15% drop in tobacco use in the last 30 years) with even greater declines in men ( 28 ). Our study details a significant reduction in smoking, and this reduction could be implicated in lower CAS values in GO patients. Furthermore, tobacco use may have influenced the risk of developing GO as well as the risk of larger goiters ( 29 ), it has been described an increase of three milliliter (mL) in male and one ml in female in thyroid volume in smoking patients ( 29 ), and a more difficult control of hyperthyroidism in these patients ( 30 ). The smaller goiter and the changes in GO described here could be related to the decrease in tobacco use in the Brazilian population or less severe hyperthyroidism at diagnosis.

The long-term supplementation of iodine is linked to a decrease in thyrotoxicosis over time despite an initial increase in hyperthyroidism ( 31 ). While data on iodine status in Brazil is not very extensive, this study was conducted in an iodine-sufficient region, where the urinary iodide excretion is around 275 μg/L ( 32 ), and seems that no significant changes occurred during this period ( 33 ).These data indicated that iodine should not be considered as the responsible agent for the mild reduced intensity in thyrotoxicosis observed in our study over the 30 years.

The new approach to public healthcare utilized by the Brazilian government created a structural hierarchy that allowed a shift in investments, with primary and secondary care receiving more founding in later years. This implies that Brazilians have better access to physicians for diagnosis and screening ( 34 ). A more efficient healthcare system could explain the phenotype presented by our patients whereas the earlier diagnosis of hyperthyroidism and a faster medical referral could be implicated in less involvement in orbital tissue and a smaller thyroid volume. Our study has several potential limitations. Firstly, our study was retrospective, and important confounders may also have been unmeasured. A second limitation is the possibility of lack of comparability among the serum determination of FT4, TSH and thyroid antibodies over these years. Despite this, we kept our primary diagnosis of GD using most accuracy criterion in the data we used. Finally, the lack of iodine excretion measurements could have some influence in our findings

In conclusion, this single university center study showed that new patients with GD have a decrease in severity in all the components of the Merseburg triad at diagnosis. This is associated with an increase in the age at diagnosis. The data showed that Brazilian GD patients are similar to those at other centers and highlight a global trend. The implications of our results need to be elucidated. The clinical characteristics of GD and GO changed in the last 30 years resulting in an increase in age, less aggressive GO, and smaller goiter. Thus, a practical therapeutic approach is needed including small doses of antithyroid drugs as a first choice and lower uses of radioiodine therapy.
